# *Physcomitrium patens* Mutants in Auxin Conjugating GH3 Proteins Show Salt Stress Tolerance but Auxin Homeostasis Is Not Involved in Regulation of Oxidative Stress Factors

**DOI:** 10.3390/plants10071398

**Published:** 2021-07-08

**Authors:** Haniyeh Koochak, Jutta Ludwig-Müller

**Affiliations:** 1 Institut für Botanik, Technische Universität Dresden, 01062 Dresden, Germany; haniyeh.koochak@wsu.edu; 2 Institute of Biological Chemistry, Washington State University, Pullman, WA 99164-5910, USA

**Keywords:** auxin conjugation, moss, oxidative stress markers, *Physcomitrium patens*, salinity stress

## Abstract

Salt stress is among the most challenging abiotic stress situations that a plant can experience. High salt levels do not only occur in areas with obvious salty water, but also during drought periods where salt accumulates in the soil. The moss *Physcomitrium patens* became a model for studying abiotic stress in non-vascular plants. Here, we show that high salt concentrations can be tolerated in vitro, and that auxin homeostasis is connected to the performance of *P. patens* under these stress conditions. The auxin levels can be regulated by conjugating IAA to amino acids by two members of the family of GH3 protein auxin amino acid-synthetases that are present in *P. patens*. Double *GH3* gene knock-out mutants were more tolerant to high salt concentrations. Furthermore, free IAA levels were differentially altered during the time points investigated. Since, among the mutant lines, an increase in IAA on at least one NaCl concentration tested was observed, we treated wild type (WT) plants concomitantly with NaCl and IAA. This experiment showed that the salt tolerance to 100 mM NaCl together with 1 and 10 µM IAA was enhanced during the earlier time points. This is an additional indication that the high IAA levels in the double GH3-KO lines could be responsible for survival in high salt conditions. While the high salt concentrations induced several selected stress metabolites including phenols, flavonoids, and enzymes such as peroxidase and superoxide dismutase, the GH3-KO genotype did not generally participate in this upregulation. While we showed that the GH3 double KO mutants were more tolerant of high (250 mM) NaCl concentrations, the altered auxin homeostasis was not directly involved in the upregulation of stress metabolites.

## 1. Introduction

Salt stress is among the most challenging situations in many areas worldwide for plant growth and development [1] since it results in both osmotic and drought stress conditions for a plant [2]. In general, mosses seem to be quite tolerant to salt stress. The moss *Physcomitrium* (recently renamed from *Physcomitrella* [3]) *patens* has become a model to study abiotic stress situations in non-vascular plants [4,5]. Drought stress responses have been studied extensively including the role of abscisic acid (ABA) [4]. In addition, it was shown that *P. patens* is influenced by salt [4] and high temperature [6], both showing cross-talk between the signaling molecules and also biotic stress factors [7,8]. The abiotic stress response of *P. patens* has been investigated on the transcriptome [4,9,10], proteome [11], and metabolome level [5]. Studies report that *P. patens* survives moderate osmotic and salt stress [4,12], and this tolerance is similar to that found for other moss species such as *Bryum argenteum* and *Atrichum undulatum* [13], but higher compared to, for example, ferns [13] or the higher plant model *Arabidopsis thaliana* [4]. Besides ABA, the auxin indole-3-acetic acid (IAA), plays a role in the salt stress response of plants [14]. During the plant life cycle, auxin is involved in a variety of growth responses and developmental processes, which are regulated in a concentration dependent manner involving the formation of auxin conjugates [15]. Conjugate formation is catalyzed by a family of IAA amino acid conjugate synthetases, of which *P. patens* has two members [16]. IAA conjugates and their hydrolysis might play a role in abiotic stress responses [15]. For example, in *P. patens* light and high temperatures adversely regulate the response in GH3 double KO mutants [17]. During salt stress, this has not yet been studied in *P. patens*, but a role for auxin conjugates has been reported in *Populus tremula* [18], *Brassica rapa* [19], and *A. thaliana*, and in the latter the overexpression of the *P. tremula* auxin conjugate hydrolase rendered the plants more tolerant of high salt [20].

The regulation of the IAA homeostasis in non-vascular plants seems to be dependent on synthesis, degradation, and to lesser extent also conjugation with small molecules resulting in inactivation [21,22], while in vascular plants the conjugation and hydrolysis of the conjugates contribute to auxin homeostasis to a larger extent [15]. In *P. patens*, the conjugation has been described [16] and it seems that it also has the capability to hydrolyze the conjugates back to free IAA [23]. However, several lines of evidence presented point to a horizontal gene transfer from bacteria for the genes identified so far [23]. In liverworts, conjugation and hydrolysis is most likely taking place already via conjugation and hydrolysis, according to the genes annotated in the respective genomes [24,25]. In contrast to the hydrolase present in *P. patens*, in *Marchantia polymorpha* a precursor enzyme of the vascular plant type has been identified [25]. Which conjugates are present in *P. patens* has, to our knowledge, not yet been elucidated, but for other moss species the occurrence of mainly IAA-Aspartate in low concentrations was described [26]. The conjugates present in *P. patens* may be inferred from by the substrates used and conjugates made in vitro and in planta [16]. While *PpGH3-1* conjugated only two amino acids (alanine and aspartate) to IAA, *PpGH3-2* had a broader substrate range in terms of amino acids. In planta the conversion of IAA added to the cultures resulting in the synthesis of IAA-Valine, IAA-Phenylalanine, and IAA-Leucine/Isoleucine, but not of conjugates with alanine or aspartate [16]. The hydrolases were able to cleave IAA conjugates with alanine, glycine, leucine/isoleucine, phenylalanine, and to a small extent valine in vitro [23], so the capability to synthesize and cleave IAA conjugates in *P. patens* seems partially to match. In planta IAA-Alanine and IAA-Glycine were cleaved, but since these hydrolases have not been expressed in publicly available databases, their role is still enigmatic.

In addition, a biosynthetic pathway, most likely via indole-3-pyruvic acid (IPyA) as the main intermediate [27], was reported due to the presence of genes known from *A. thaliana* to convert IPyA (TAA, YUCCA) [28]. The transport of IAA in mosses such as *P. patens* was described in [29] based on the occurrence of genes encoding influx [28] and efflux carrier proteins [30]. Export would result in a direct reduction through the small tissue layers of, e.g., leaves [31]. The existence of a degradation pathway has also to be considered, especially since Stzein et al. [21] indicated a degradation pathway for IAA. The existence of proven IAA oxidase enzymes in *A. thaliana* [32,33] will make it easier to identify whether these are present in *P. patens* or if another strategy to degrade IAA is used.

Other typical stress metabolites include sugars such as trehalose [5], phenols [20], flavonoids [34,35], ROS and ROS detoxifying enzymes such as peroxidase (POX) and superoxide dismutase (SOD) [36]. In general, the phenolic and flavonoid biosynthetic pathways are considered conserved in nature, therefore mosses already make use of these stress compounds [37,38]. In *P. patens*, for example, UV-B treatment can induce flavonoid biosynthesis [38]. Among the phenols, phenolic acids can accumulate under salt stress and detoxify ROS due to their antioxidative potential [20]. Additionally, lipid peroxidation as a result of the presence of ROS can be determined as the amount of malondialdehyde present in tissues [19]. While there are many roles assigned to peroxidase isoenzymes, one major one is in stress protection [39]. Both antioxidative enzymes POX and SOD are important for the scavenging of ROS [34,39].

Many studies report that *P. patens* survives osmotic and salt stress (e.g., [4,12]). Therefore, the aim of this study was to continue the investigation into the role of auxin homeostasis in *P. patens* by using the previously published GH3 double KO lines [16] and expose these together with WT to different salt concentrations. This should broaden the knowledge already available for *P. patens* under salt stress [4,5,6,9,12], but focusing on a possible role for auxin in this scenario. Since all mutants were generated based on homologous recombination and are all true knockout lines [16], the effects that we observed might be due to alterations in the lines due to possible long cultivation periods. While we showed that the GH3 double KO mutants were more tolerant concerning some phenotypical parameters at high (250 mM) NaCl concentrations, the altered auxin homeostasis was not directly involved in the upregulation of stress.

## 2. Results

### 2.1. Growth of Physcomitrium patens at High Salt Concentrations and Revival

**Figure 1:**
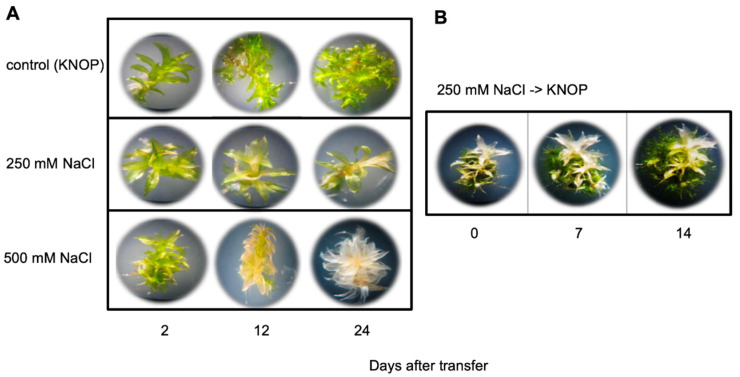
Growth of *P. patens* gametophores on medium containing high salt concentrations: (**A**) Individual gametophores are shown that have developed for 2, 12, and 24 days on either control medium without salt or on 250 mM and 500 mM NaCl; (**B**) Individual gametophores were transferred after 3 weeks from 250 mM NaCl to medium without salt and were photographed 7 and 14 days after transfer. In panel (**B**), at each time point the same plant was photographed.

**Figure 2:**
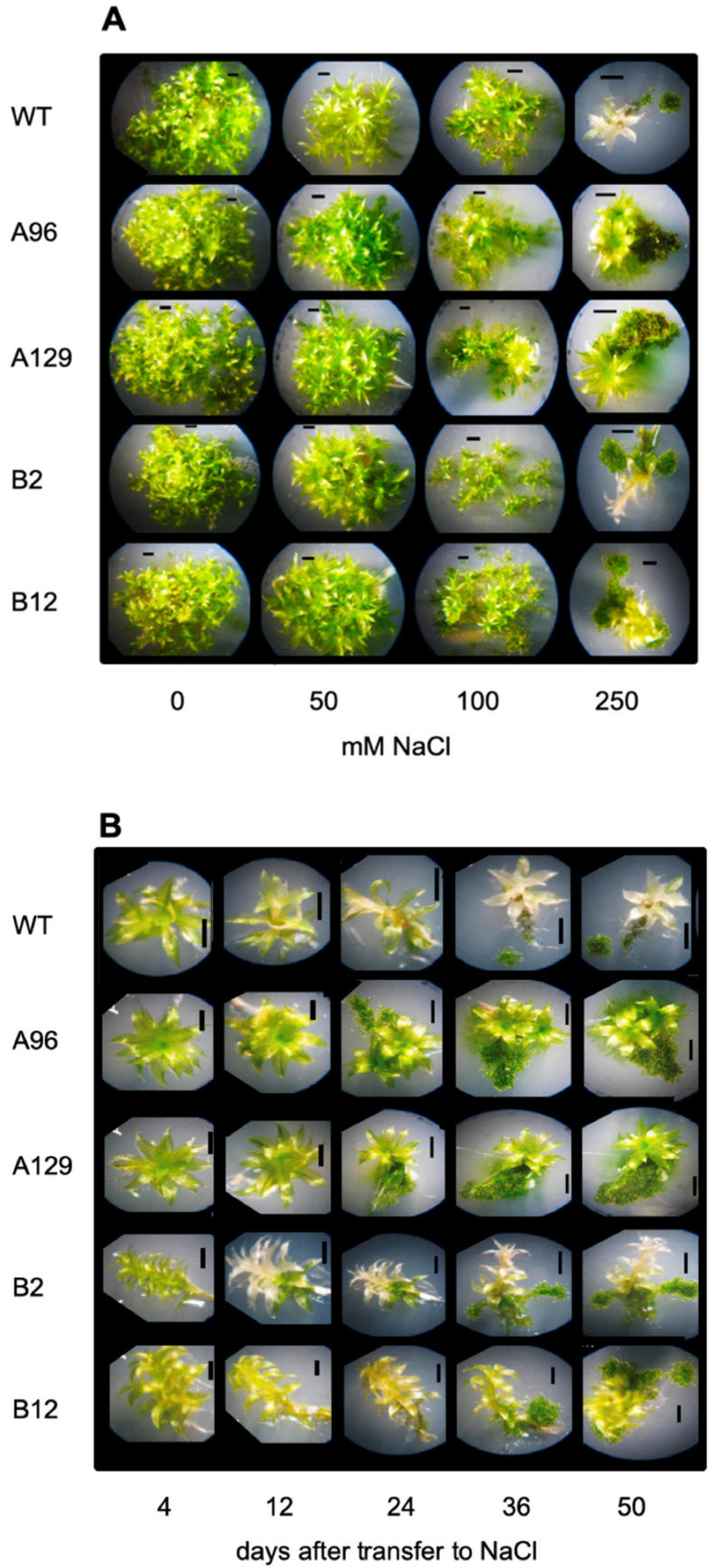
The growth of *P. patens* WT and four different double knock-out mutants in the two *GH3* genes:.(**A**) Different NaCl concentrations 50 days after transfer to salt-containing media. (**B**) Time course on 250 mM NaCl. Selected colonies are shown here in magnification. The pictures of the complete plates under the same conditions are shown in the supplement.

One hundred mM NaCl is considered moderate salt stress in *P. patens* since exclusion of Na via sodium ATPase (PpENA1) ensures normal growth of *P. patens* under moderate salt stress [40], but higher concentrations in experiments with *P. patens* have also been reported [4]. To set up a test system, we have included not only low, but also very high salt concentrations. At 500 mM NaCl, bleaching of leaves was observed three weeks after transfer to salt medium (Figure 1A), whereas at 250 mM NaCl the plants were still viable after even 50 days (see also Figure 2). However, this was mainly due to green protonema, whereas the gametophores also showed bleached leaves. Therefore, we hypothesize that the protonema tissue is less sensitive to high salt concentration and might be regarded as the duration tissue. It was possible to revive the gametophores from 250 mM NaCl (Figure 1B), but not from 500 mM (data not shown). Three weeks after transfer to control media, new gametophores developed and were green. The gametophores showed survival at up to 500 mM NaCl, at least for two weeks (Figure 1A). The survival was for a longer time period (up to 50 days after transfer colonies were still green, even though they did not show any more growth; Supplementary Materials Figure S1). Individual gametophores showed greening of the formerly whitish parts.

### 2.2. Digital Expression Analysis Showed That P. patens GH3 Genes Are Differentially Expressed during Abiotic Stress

**Figure 3:**
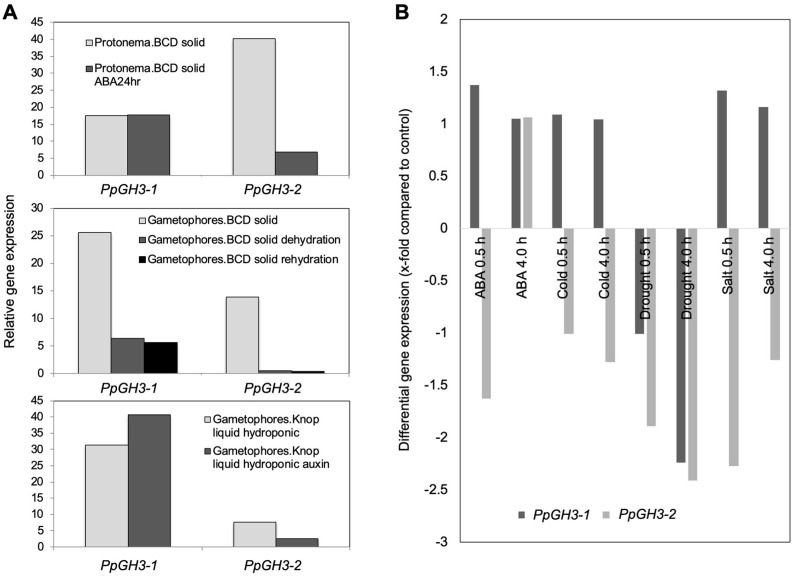
Selected samples from digital gene expression analysis of both *GH3* genes using published data from Gene Atlas (Phytozome) [41,42] (**A**) and eFP browser [43,44] (**B**).

Digital transcription analysis of the two *PpGH3* genes under various stress conditions was monitored using available web resources (Phytozome, GeneAtlas, Thermo Fisher Scientific, Dreieich, Germany) [41,42] and GFP browser [43,44]. Since desiccation tolerance can be induced by exogenous ABA [45,46], ABA treatment was also included in this analysis. Upon dehydration, both *GH3* genes are strongly downregulated in gametophore tissues, but ABA treatment resulted in the upregulation of *PpGH3-2*, but not *PpGH3-1* in protonema cultures (Figure 3A). This difference could possibly be explained by a promoter element present that would not respond to ABA. Dehydration resulted in a down-regulation of both *PpGH3* gene transcripts, whereas salt stress only led to a downregulation of *PpGH3-2* in a different web-resource (Figure 3B). In addition, the GH3 genes from *P. patens* are also developmentally regulated (data not shown; from [43,44]).

The promoter sequences of both *GH3* genes were analyzed for the occurrence of possible regulatory elements circa 3 kb upstream of the transcription start, but regulatory elements such as TATA- or CAAT-boxes were not included (Table S1; from Phytozome [40]. Function of the element and element names are given, as well as the number of the elements in the respective promoter region (in brackets). Only the promoter of *PpGH3-1* has elements that were assigned to ABA inducibility. Both have an element that is assigned to be responsible upon drought. Interestingly, the promoters both do not have obvious predicted auxin-response elements. This is in accordance with digital gene expression levels of gametophores after auxin treatment (Figure 3A).

### 2.3. PpGH3 Double KO Mutants Show More Tolerance to Growth on High Salt

Since it is known that IAA homeostasis plays a role in salt stress responses in different plant species such as *A. thaliana* and *P. tremula* [17], and the GH3 proteins of *P. patens* seem to be involved in other stress responses, we investigated whether the previously described double knock-out mutants [7,15,16] show altered phenotypes in comparison to WT. The gametophore phenotype after salt stress was analyzed and it was found that the gametophores of all mutant lines showed a retardation in bleaching of the leaves at higher NaCl concentrations. In addition, the protonema was taking up larger areas on the plates, so that the total growth diameter of the mutants was higher than WT (Figure 2). All GH3 double mutants were more tolerant to salt stress as shown after 50 days of transfer to 250 mM NaCl (note the WT photo is the same as in Figure 1).

**Figure 4:**
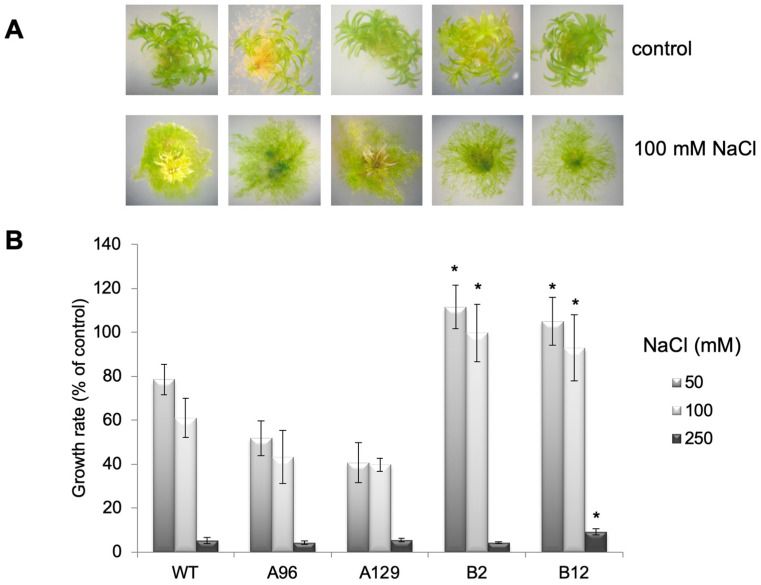
Growth of protonema is promoted on high salt concentrations, which results in accelerated growth rates: (**A**) Examples of WT and GH3 double KO lines on control medium and medium containing 100 mM NaCl; (**B**) The growth rate of the same lines on 50, 100, and 250 mM NaCl determined as percent of the control without NaCl over the time of 50 days after transfer to salt-containing medium. The asterisks indicate significant differences between WT and mutant lines at *p* < 0.05.

In comparison to the scattered growing protonema on 100 mM NaCl, the colonies on 250 mM NaCl have darker green, smaller, and denser protonema with a slower growth rate. The mutant lines in particular show an extended protonema growth compared to WT (Figure 2), which is indicative of a possible protective role for the protonema tissue for stress tolerance. However, the fresh weight under these conditions was not significantly different between WT and the mutant lines (Figure S2). A more detailed analysis including the growth rate showed that especially two lines were significantly more tolerant while two others were not (Figure 4). Since the mutant lines are all generated via homologous recombination, differences between lines cannot be explained by the genetic background, but rather by alterations that must have occurred during the long cultivation period between mutant generation and experimental period. In future experiments this can be addressed by, e.g., transcriptome analyses.

The water loss calculated from the ratio dry weight to fresh weight was also not significantly changed when WT was compared with two mutant lines at different salt concentrations (Figure S3). These observations confirm that the loss in auxin conjugation does not alter growth per se, but the ratio of gametophytes vs. protonema might be altered. This supports the observation that the protonema tissue mainly responded to salt stress, which is a minor fraction of the weight of the plant. The quantification of protonema growth alone is difficult due to the irregular shape of the colonies.

**Figure 5:**
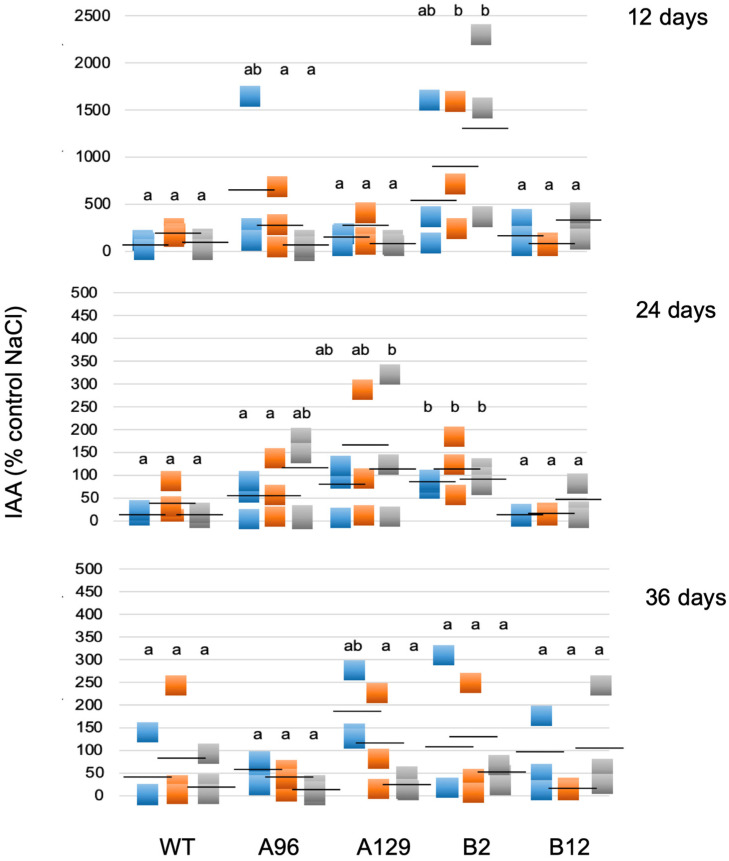
Free IAA levels given as % on control medium (0 mM NaCl). Blue = 50 mM NaCl; orange = 100 mM NaCl; grey = 250 mM NaCl. The IAA was determined at three different time points for three replicates. Different letters indicate significant differences to the control without NaCl at *p* < 0.05. Each square represents an individual data point and the black dash represents the mean value.

In addition, free IAA was determined in the samples grown on 0, 50, 100, and 250 mM NaCl over a period of 12 to 36 days after transferring gametophores from control medium. The data were expressed on the basis of the control treatment (0 mM NaCl) for each line and time point. Using this type of calculation, the differences for the individual lines can be better seen. While the replicates had high variations, nevertheless differences in free IAA were detectable. During the course of the experiment (12, 24, and 36 days at 50, 100, and 250 mM NaCl) an increase in the free IAA content at 12 days was observed only for one KO line, but after 24 days three out of four mutant lines showed a significant difference to WT in the IAA levels for at least one salt concentration, while after 36 days the effect was no longer significantly different (Figure 5). Therefore, the increase in free IAA might be one of the factors responsible for the tolerance of the GH3 double-KO lines. Additional possibilities would be that higher auxin levels are indirectly responsible for the tolerant phenotype of the mutants as a result of a delay in senescence. The greener leaves of mutant lines compared to WT at high salt levels would fit into this hypothesis (Figure 2).

**Figure 6:**
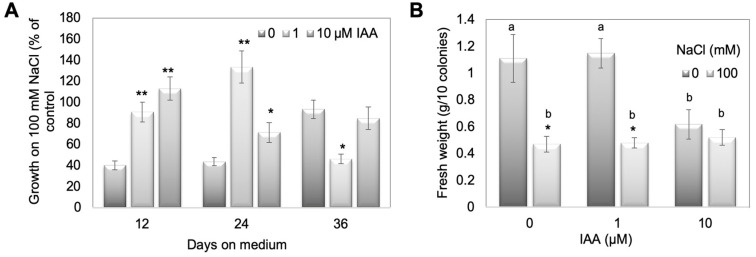
IAA (1 and 10 µM) treatment of WT together with high salt concentration (100 mM). (**A**) Growth rate as percent of control between 0 and 36 days given as diameter of three time intervals at which the measurements were done and (**B**) fresh weight after 36 days was recorded. In (**A**,**B**), the mean values for 12 plates with 10 colonies on each plate are given. Asterisks in (**A**) denote statistically significant differences between control and IAA treatments on 100 mM NaCl expressed as percentage of the control, in (**B**) denote statistically significant differences between control and NaCl treatment (* *p* < 0.05, ** *p* < 0.01), and different letters in (**B**) indicate differences between control and IAA treatments. Calculations for growth rate in (**A**) were done by measuring the diameter of colonies at 0 days (day of transplanting on fresh medium), diameter of colonies at 12, 24, and 36 days on treatment medium and subtracting the respective later time point from the earlier one. The value for 12 days is thus the difference/growth rate between day 0 and day 12, the value for 24 days growth between 12 and 24 days, and the value for 36 days represents the growth between 24 and 36 days. To show the effect of NaCl compared to control, for each value +/− IAA the percentage of growth on 100 mM NaCl compared to the control was calculated. Values higher than the control value without IAA are an indication that the moss plants are performing better on simultaneous NaCl and IAA treatments.

To test this assumption, WT plants were treated with 100 mM NaCl, where the growth was positively affected for two mutant lines (Figure 4), concomitantly with two different IAA concentrations (1 and 10 µM). Since free IAA levels were under some conditions higher on salt in the mutants compared to WT, this experiment should mimic the conditions in the mutant lines. The treatment of WT moss with 1 and 10 µM IAA showed that the growth phenotype could be phenocopied to some extent (Figure 6). The growth rate as a percent of control between 0 and 36 days was determined as the diameter of three time intervals at which the measurements were done (Figure 6A) and the fresh weight of all colonies was determined after 36 days on media (Figure 6B). Based on the calculations described in detail in the legend to Figure 6, values for the IAA treatments that are higher than the control value are an indication of the moss plants performing better on simultaneous NaCl and IAA treatments. For the first two time points there were differences between WT with NaCl and IAA compared to WT with only NaCl. However, this trend was not visible any more at later times, which ended after 36 days with NaCl and for which the fresh weight was also determined. Like for the resistant mutant lines (Figure S2), the fresh weight was not an indication of stress tolerance. This might again be due to the involvement of protonema in growth responses, which do not contribute significantly to the weight of the overall culture (Figure 4 and Figure 6).

### 2.4. Salt Stress Upregulates Different Stress Parameters in Gametophores, but PpGH3 Proteins Are Not Involved in Their Upregulation

The response to abiotic stress involves an increase in low molecular weight scavenging molecules and enzymes since the stress might result in the generation of reactive oxygen species (ROS). Therefore, different stress-related parameters were assessed to evaluate which stress response might be upregulated in mutants compared to WT. Here, total phenols, lipid peroxidation, flavonoids, as well as peroxidase and superoxide dismutase enzyme activities were tested. Flavonoids are not only stress-induced compounds, but also modulate auxin transport [47]. We found, with very few exceptions, no genotype specific differences between WT and mutants, but differences in the WT response to salt stress were observed. In addition, twelve days after treatment not many effects were found, so the data are in most cases given for 24 and/or 36 days after transfer to salt-containing media. The complete dataset for all time points, NaCl concentrations, and genotypes can be found in the Supplementary Materials (Figures S4, S6 and S7).

**Figure 7:**
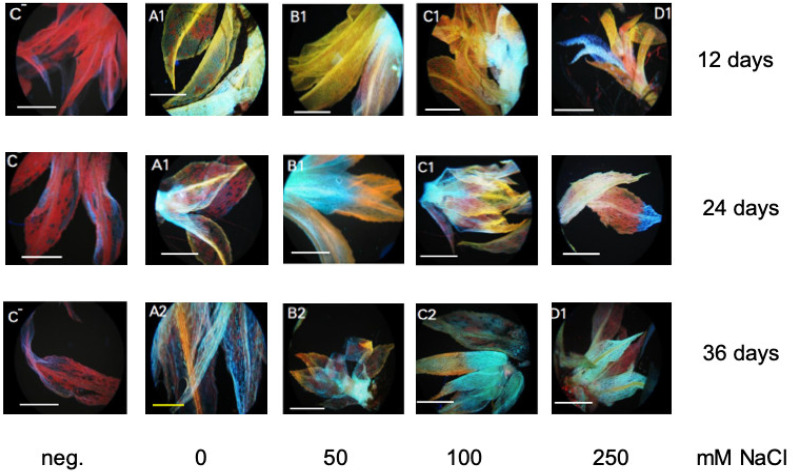
In situ staining of flavonoids with DPBA according to the method of Buer and Muday [53]. Individual gametophores are shown for the different salt concentrations at three different time points. The first panel is the negative (neg.) control without DPBA (C^−^); mainly showing the red autofluorescence of chlorophyll. Yellow color denotes quercetin derivatives, cyan naringenin derivatives, and green kaempferol derivatives, while blue denotes sinapate derivatives. The negative controls without staining solution show only the red chlorophyll fluorescence. Only one set of pictures for WT is shown for all three time points. All data including two mutant lines are shown in the Supplementary Materials (Figure S6). The bars indicate 100 µm.

Bryophytes contain only flavonoids synthesized early in the phenylpropanoid pathway, that is naringenin chalcone and naringenin, dihydrokaempferol and kaempferol, and dihydroquercetin and quercetin [48,49,50]. A gene for narigenin chalcone synthesis is annotated in the KEGG (Kyoto Encyclopedia of Genes and Genomes, Kyoto, Japan) database for *P. patens* (Figure S5). During stress response (UV light), a band corresponding to a quercetin derivative was detected in *P. patens* leaves by thin layer chromatography [34]. Flavonoids were determined in this work also with diphenylboric acid 2-amino-ethyl-ester (DPBA) that binds to flavonoids enabling their in situ visualization. The DPBA-flavonoid complex will show a specific fluorescence color depending on the structure of the respective flavonoid and is therefore specific [51]. Since there were no differences between the mutant lines and WT, only WT leaves are shown for three different NaCl concentrations for all three time points (Figure 7). Two gametophores were randomly picked for the photographs (Figure 7). The yellow-orange colored leaves contain quercetin and Q-derivatives, such as the glycoside rutin [51,52,53]. The cyan color stands for naringenin, green for kaempferol, blue for sinapate derivatives, and red is the chlorophyll autofluorescence [51,52,53]. The group of quercetin derivatives seems to decrease over time and with salt stress. Naringenin is present in all samples, whereas kaempferol could only be detected in a few spots. Sinapate derivatives are more present at higher salt concentrations.

**Figure 8:**
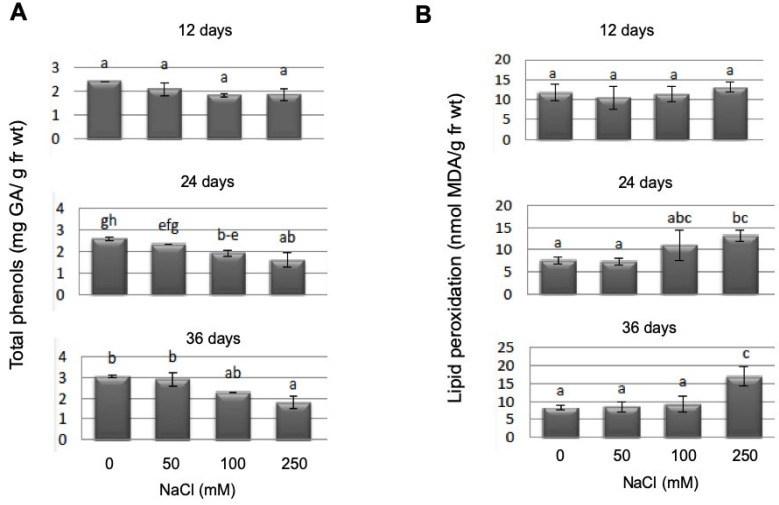
Total phenols determined as gallic acid equivalents (**A**) and lipid peroxidation as determined as MDA equivalents (**B**) determined in WT gametophores at different salt concentrations and at three different time points. Bars with different letters are significantly different at *p* < 0.05. Since the mutant lines did not show any difference in WT in their response to salt stress with these parameters, the complete dataset is displayed in the supplement.

Total phenols showed a significant reduction under salt stress (between controls and 250 mM NaCl), but only at later time points (Figure 8A). Lipid peroxidation as a marker for membrane stress was significantly upregulated in *P. patens* gametophores after salt stress (250 mM NaCl), again at the two later time points (Figure 8B). As expected, the longer the gametophores remained in high salt conditions, the more lipid peroxidation could be detected.

**Figure 9:**
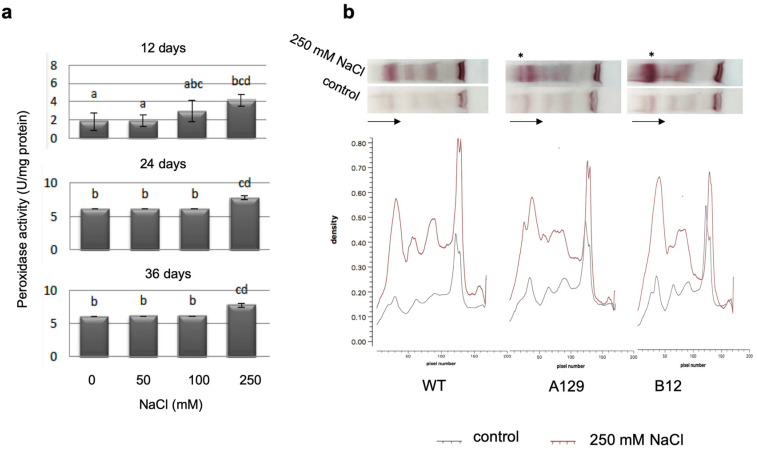
Peroxidase activity of WT determined with OPD (**a**) and isoenzyme separation of WT and two mutant lines on native polyacrylamide gels using benzidine-guaiacol for visualization (**b**). Bars with different letters are significantly different at *p* < 0.05. Since the mutant lines did not show any difference to WT in their response to salt stress with the parameters determined in (**a**), the complete dataset including the gel stained with Coomassie blue is displayed in the Supplementary Materials (Figures S4 and S7). The density of the bands in (**b**) was detected using the GelEval software free trial version 1.35 (FrogDanceSoftware, Cambridge, UK). The asterisks mark differences between WT and mutant lines in the peroxidase isoenzyme pattern. The arrows show the direction of sample flow.

For total peroxidase activity, significant differences after 250 mM NaCl treatment were found, which persisted over all measured time points (Figure 9a). Analysis of specific peroxidase isoforms using a native PAGE method [54] showed some quantitative differences between the treated WT and mutant plants, being the only detectable differences in the whole dataset (Figure 9b). All bands increased in intensity in the salt treated samples of WT and mutants, and an increase in a set of isoenzymes with high molecular mass (marked by asterisks) was found that was higher in the two mutant lines than in the WT. The complete dataset is shown in the Supplementary Materilas (Figure S6).

**Figure 10:**
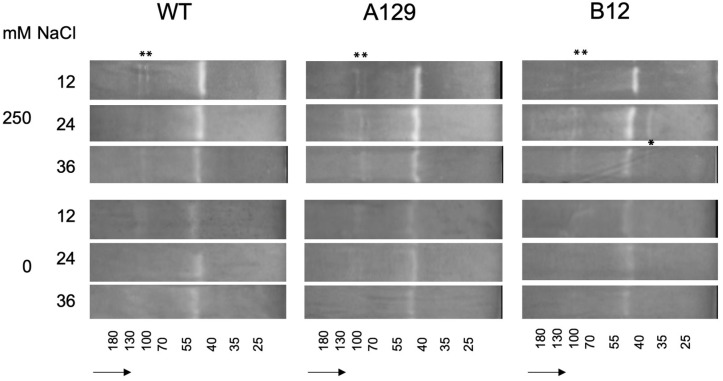
Superoxide dismutate (SOD) isoenzyme separation shown for two selected mutant lines in comparison to WT. Asterisks mark differences between WT and mutant control samples and 250 mM NaCl. For mutant line B12 an additional band is marked. The complete gels including the gel stained with Coomassie blue are shown in the Supplementary Materials (Figure S6). The small numbers at the bottom indicate the approximate molecular masses from marker mixtures. The arrows show the direction of sample flow.

For superoxide dismutase the differences in isoenzyme patterns that can also be visualized on native PAGE by activity stain [55,56] were mainly visible between control and salt treatments, but not between genotypes (Figure 10). There were only slight increases in three different bands and one additional band was found in one of the mutants (B12) (marked by asterisks). The complete gel pictures including the Coomassie stained gel for total protein are shown in the Supplementary Materials (Figure S7).

## 3. Discussion

The majority of plant species cannot endure high concentrations of salt, which causes ion imbalance and hyperosmotic stress and adverse effects following that. In comparison with other plants such as *A. thaliana*, *P. patens* exhibits a high degree of abiotic stress tolerance. This characteristic makes it invaluable for use in investigations into the identification of genes related to stress adaptation [4]. The identification of salt signaling components and transcription factors in bryophytes suggested that salt stress can lead to activation of adaptive responses and that bryophytes are able to tolerate salt stress by means of multiple biochemical pathways [4,9,11,46]. The salt tolerance of *P. patens* is high compared to other plant species [4].

The work presented here indicates the involvement of auxin homeostasis in the salt tolerance response of *P. patens*. The control of auxin levels is very important for all developmental processes in land plants, and also abiotic stress responses [14]. It is mediated, among other important mechanisms, by the conjugation of the free active hormone to amino acids catalyzed by auxin amino acid conjugate synthetases from the GH3 family [14,57]. Among the abiotic stressors, salt stress is a complex trait that results in many different branches within the response. For example, it affects the osmotic homeostasis and parts of drought stress responses due to reduced water uptake [58]. In addition, the upregulation of some “classical” responses to abiotic stress such as phenols, lipid peroxidation, peroxidase, superoxide dismutase needs to be considered [19,54,55,56]. However, except for a few data, no correlation was found between the knock-out of auxin conjugation and the induction of stress parameters after growth of moss gametophores with high salt levels. Several studies have reported on free IAA levels and also IAA conjugates during salt stress. In several plant species, such as rice, tomato, and wheat, IAA was reduced [59,60,61], whereas IAA amino acid conjugates were upregulated in other plant species [62]. However, there are also reports on the contrary response, i.e., in *Brassica rapa* free IAA was upregulated and some conjugates decreased [63]. Such findings could be confirmed on the transcript level [18,58,64,65]. If one could reverse these IAA vs. IAA conjugate levels by introducing or knocking down genes involved in the conjugation/hydrolysis of IAA conjugates, the IAA would increase, and amino acid conjugates decrease in such a mutant. In *A. thaliana* the increase in IAA via overexpression of an IAA conjugate hydrolase increased salt tolerance [17]. Therefore, the intrinsic IAA levels might be one factor to trigger salt stress tolerance. The double GH3 KO mutants of *P. patens* show the same auxin patterns, namely increased IAA and no amino acid conjugates [16]. Based on these predictions the mutant plants could be more tolerant to (salt) stress.

Indeed, a role for the auxin conjugation in *P. patens* for other abiotic [16] and biotic stressors has previously been shown [7]. High IAA levels were responsible for tolerance during growth in darkness, but on the contrary caused higher sensitivity to elevated temperature stress [16]. Biotic stressors were also affected by the higher IAA levels of the GH3 double KO lines. It was shown that the oomycete *Pythium debaryanum* inhibited growth at high IAA concentrations in vitro, so the inhibition of disease progression in the GH3-KO lines with higher IAA content could be explained by this [7]. Alternatively, the auxin effect might be indirect since auxin could delay senescence, which is corroborated by the whiter leaves of WT compared to all mutant lines at high salt concentrations (Figure 1). Previously, it was shown that the same lines showed a delay in senescence in darkness, but an accelerated senescence at higher temperatures [17].

It was shown that a large number of transcripts that respond to dehydration were differentially regulated and several a sub-population also responded to exogenous ABA [66]. Khandelwal et al. [67] demonstrated that the *P. patens* homolog of the *A. thaliana* gene *Abscisic acid Insensitive 3* (*ABI3*) is required for desiccation tolerance to be induced by the application of ABA. A microarray study using an array consisting only of transcription factors compared salt stress and ABA signaling in *P. patens* [9]. In mosses, ABA and drought induce the differentiation of protonema cells into brachycytes (brood cells), which represent vegetative propagules for survival under adverse environmental conditions [46]. The transcriptional regulation, as found in public transcriptome datasets, of both *GH3* genes (Figure 3) suggested a possible role within drought or salt stress as well as after treatment with ABA. In addition, the promoters contain several elements related to upregulation of stress (Table S1). We have therefore analyzed the stress tolerance of WT and GH3 double KO mutant lines under different salt concentrations (moderate to very high).

While investigations with WT are often conducted [4,5,6,9,11,12], there is no information as to whether there is a direct connection between auxin and salt stress in *P. patens*. For *A. thaliana* and other higher plants there is more information [14], for example it was found that overexpression of a poplar auxin amino acid conjugate hydrolase resulted in more salt tolerant plants [17]. The hydrolysis of IAA conjugates also results in the increase of free IAA. Likewise, the GH3 double KO mutants have higher IAA levels when grown on medium supplemented with IAA [15] and under stress [16]. Earlier it was reported that in vitro-cultured moss that were exposed for three days to medium containing increasing concentrations up to 350 mM NaCl were able to recover themselves, while the samples at 500 mM NaCl were completely bleached two weeks after treatment [4]. Under our growth conditions, a similar trend, namely the bleaching of samples two weeks after transfer to 500 mM NaCl and their disability to recover themselves after moving to non-stress conditions was confirmed (Figure 1 and data not shown), while growth at 250 mM NaCl enabled the moss to be revived, but the colonies were also bleached after longer cultivation times at high NaCl levels (Figure 1). In addition, all mutant lines looked greener at high NaCl concentrations for a longer growth period and two showed better growth rates (Figure 3, Figure 4 and Figure S1). The data presented here confirm that the mutant plants are more tolerant to high salt concentrations, and that this was mainly due to the capacity of the protonema to grow further (Figure 3 and Figure 4). Previous work has already pointed out the significant role of protonema in abiotic stress response tolerance (reviewed in [54]). However, the growth effect could only be quantified for the two lines B2 and B12, not for the A mutant lines (Figure 4). Since both mutants are generated by the homologous recombination technology, they are true knockouts without any residual transcriptional activity [15]. Different effects between lines could therefore also not be explained by insertion effects. A possibility exists that other events occurred in these lines independent of the primary mutation event and are not directly connected to the primary phenotype. We have to assume that these line specific effects might have been caused by later changes during the clonal cultivation period of these plants.

Since in the GH3 double KO lines a higher IAA content was described under control conditions [15,16], we hypothesize that the elevated IAA levels could be beneficiaries of stress, so that the respective colonies showed better growth at high salt levels. However, under normal conditions the high IAA is not beneficial for growth [15], and this is the reason why it needs to be tightly controlled by the GH3 proteins also in *P. patens*. Other stress factors, light and elevated temperature, also resulted in increased free IAA in two GH3 double KO lines as shown in previous work [16]. However, the extent to which that occurs seems to be dependent on the type of stress, so the increase observed for salt treatment could differ for other stressors. The mutant plants were more tolerant at higher temperatures, but not to growth in darkness [16]. When free IAA levels in the mutants were compared under different salt concentrations to WT, a relative increase was found between salt and control medium that increased after 12 days only for one mutant line, after 24 days more lines, while 36 days after treatment the effect was not significant any more (Figure 5). Under salt stress conditions, treatment of WT gametophores concomitantly with IAA and NaCl resulted in the approximate phenocopy of the mutant growth behavior (Figure 6). While the fresh weight did again not reflect any changes, the colony diameter of those WT plants treated with 1 and 10 µM IAA was higher with 100 mM NaCl compared to the colonies grown without IAA. The increased IAA levels in the GH3 double-KO lines could thus be one reason for their survival with salt. Additionally, in this work, the protonemal growth was in part responsible for the observed phenotype (Figure 3, Figure 4 and Figure 6). The determination of the widest point of the diameter might not exactly reflect colony size, so using additional digital software to determine the complete gametophyte area could be useful for future research. Previous publications illustrate that the application of auxin to *P. patens* results in a faster transition from chloronema to caulonema and further maintenance of the desired concentration arrests the development in the caulonema stage [68]. In addition to chloronemal branching, this treatment converts most gametophore tissue into rhizoid cells [69,70]. This in turn provides a sufficiently dense protonemal mat that maintains a moist soil (or medium) surface [71]. These observations make auxin an important regulator of this developmental transition and some genes responsible for this transition have been identified [72]. Previous work has also shown that the auxin response under stress was at least partially linked to protonema filaments [16]. Together with our observations for salt stress we can confirm the potential of protonema in the stress tolerance [68,69,70].

There are other examples where higher conjugation levels, i.e., to the auxin indole-3-butyric acid (IBA) in *A. thaliana* resulted in more stress tolerant plants [73]. On the contrary, NaCl could also directly reduce IAA levels, e.g., in tomato via an ABA-independent pathway [74]. As mentioned above, in *A. thaliana*, and other higher plant species, the hydrolysis of auxin conjugates plays a role in homeostasis and stress responses [14,17,18]. While the GH3 proteins of *P. patens* are similar in sequence and function to the higher plant enzymes [15], the hydrolysis pathway was thought not to take place at all, since the typical plant sequences for conjugate hydrolases were missing [75]. However, recent work provided experimental evidence for the possible hydrolysis of IAA amino acid conjugates in *P. patens* as well, since it was found that the moss contained sequences similar to bacterial auxin amidohydrolases [24,76] and its capacity to cleave some conjugates in vivo and in vitro [24]. These sequences were possibly acquired by horizontal gene transfer [76]. Modeling of one protein sequence showed similarity to the respective proteins from *A. thaliana* [76]. Although as far as we know no one has examined *P. patens* to determine which auxin conjugate is the native product so far, the most likely of these compounds to be present in vivo may be those described for other moss species [76]. The authors found that the most common moss conjugate was IAA-Aspartate at low concentrations. They concluded that liverworts prefer conjugation as a regulatory scheme while mosses favor degradation strategies to maintain homeostasis. This conclusion is supported by the observation that none of the aforementioned hydrolases was expressed in publicly available databases [40,41,42,43].

We conclude that auxin is mediating at least some part of the salt stress response of *P. patens*, but in almost all cases not via the upregulation of the selected stress metabolites measured in this investigation (Figures S4, S6 and S7). Salt stress can result in an increase in the amount of reactive oxygen species (ROS) within plant cells [77]. These ROS are extremely reactive and will damage different cellular parts, for instance DNA, lipids, and proteins. Any serious imbalance between ROS production and antioxidant defenses can cause oxidative stress in plant cells [77]. Therefore, being tolerant to salinity is related to their antioxidative response. Generally, tolerant plant species can produce and maintain a larger amount of antioxidant molecules and an enhancement in antioxidant enzyme activity under stress conditions, so that they will have a better function to protect against salt-induced oxidative stress damage [78]. Typical land plant stress metabolites thus include many compounds with antioxidative potential such as phenols, flavonoids, and sinapate derivatives [79,80,81], but also enzymes involved in the detoxification of ROS [54,55,56,82].

While the lipid peroxidation level increases as a response to increasing NaCl concentrations in moss gametophyte cultures (Figure 8B), we could not detect a specific response in terms of an increase in total phenols as stress metabolites, rather their concentration decreased during NaCl stress (Figure 8A). Since flavonoids are present and play a role in early land plants such as *M. polymorpha* [48], the occurrence of flavonoids in *P. patens* during salt stress was assessed. The occurrence of quercetin derivatives has been reported in a thin layer chromatography analysis after UV treatment [37]. Recent work identified *P. patens* enzymes capable of converting naringenin to apigenin and a dihydrokaempferol derivative [49]. This indicates that enzymes capable of synthesizing flavonols are present, which might in vitro not reflect the complete in planta spectrum and that the color patterns we found could indeed correspond to the flavonols is mentioned in the results. After staining of *P. patens* cultures with DPBA, a decrease over time and higher NaCl stress for putative quercetin derivatives in yellow-orange were found, while the sinapate fraction (in blue) seemed to increase over time and stress level (Figure 7), but no changes between WT and mutant lines were observed (Figure S6). Other putative flavonoid derivatives as indicated by their typical fluorescence did not show any alterations. Of course, the precise identification of these compounds needs to be achieved in future work. Both groups of compounds are discussed as stress metabolites in plants, which are altered under different stress conditions [79,80,81]. In particular, the antioxidant function of flavonoids [80] could play a role in the stress response we observed in *P. patens*, since the lipid peroxidation levels were increased under NaCl stress (Figure 8B).

The activity of enzymes involved in the antioxidative stress response such as peroxidase and superoxide dismutase [82] was also analyzed. Interestingly, there is a report on the downregulation of peroxidases by several IAA amino acid conjugates [83]. Thus, a reduction of GH3 proteins that would also result in lower levels of IAA conjugates in *P. patens* could be involved in the mediation of the level of stress enzymes. However, there was no clear link towards an increase in total peroxidase activity between WT and mutants, since in all plant lines the activity increased at high NaCl levels (Figure 9a and Figure S4). Therefore, the isoenzyme patterns were analyzed by native gel electrophoresis [54]. Here, an indication of a possible connection between GH3 double-KO lines and increased peroxidase activity was found (Figure 9b and Figure S7). In both tested mutant lines, there was an additional band visible, and one band also present in WT was considerably stronger in both mutant lines (marked by asterisks). Similarly, the isoenzyme patterns for superoxide dismutase were visualized after PAGE (Figure 10 and Figure S7). While there were NaCl-dependent changes in individual bands, only one mutant line showed a difference in WT, so it was concluded that this is most likely a line-specific effect, even though the targeted knock-out of the genes [15] in theory should not lead to such differences. The low response of SOD isoenzymes corresponds to the observation that *P. patens* double knock-out mutants for chloroplastic SOD does not show any alteration in abiotic stress tolerance, especially when the growth of protonema under high salt conditions was assayed [84].

This work has uncovered novel players in the salt stress response of *P. patens* independently of the auxin homeostasis. Browsing the available transcriptome [9,10,41,42,43,44,66] and proteome sets [11] for *P. patens* can lead to many other putative targets by analyzing the genes upregulated by osmotic and salt stress, but the analysis of end products or enzyme activities helps to identify the active components of a stress response system more directly. For example, we have analyzed the most strongly co-expressed genes from Gene Atlas (Phytozome) [41,42] with the two *PpGH3s* as query and found some candidates involved in abiotic or biotic stress responses for *PpGH3-1* (Table S2), among them two receptor protein tyrosine kinase/non-specific serine/threonine protein kinase/threonine-specific protein kinase; one WRKY DNA-binding domain). In addition, a gene encoding a TRYPTOPHAN SYNTHASE BETA CHAIN was strongly co-expressed with *PpGH3-1*. The functional analysis of such targets identified by bioinformatic methods can further contribute to the salt stress response of *P. patens*.

## 4. Materials and Methods

### 4.1. Plant Material and Experimental Conditions

The plant material (WT and 4 GH3 double KO lines) was described in Ludwig-Müller et al. [15]. The moss cultivation was performed according to Mittag et al. [16] on Knop medium supplemented with 1.2% agar. Single gametophores were placed onto the plates (10 per plate) at the beginning of the experiment using forceps. Plates were sealed with micropore tape (3M, Neuss, Germany). Control plates did not contain any salt. Cultivation was performed in a growth chamber at 25 ± 1 °C under a 16/8 h light/dark photoperiod.

NaCl was added at the appropriate concentration (50, 100, 250, and 500 mM) to the agar plates. The respective IAA concentrations (1 and 10 µM) were added from stock solutions (IAA dissolved in ethanol, the final concentration was 1% in the agar plates after dilution) according to Ludwig-Müller et al. [15]. For revival experiments individual gametophores were removed from the NaCl-containing agar and placed on Knop agar without salt. At different time points, as indicated in the results, plants were inspected, the diameter of the colonies measured at the widest point as described in Mittag et al. [16], and if appropriate for the respective analyses the plants were harvested to determine fresh weight as well as secondary metabolites, lipid peroxidation, hormones, and enzymes as described in more detail in the following sections. The growth rate was determined by subtracting the diameter of the respective moss colony at later time points from that of an earlier time point. In some cases, the values were normalized on the untreated samples and expressed as percent of control.

For the measurement of the fresh weight, the colonies were removed thoroughly from the plates to reduce contamination with agar. A comparison between the fresh and dry weight of each sample was done on day 36 for WT and two mutant lines (A129, B12) at zero and three salt concentrations. Of each sample a representative amount that was weighed before was kept in an oven at 65 °C for 3 nights. After the samples were reduced to complete dryness, their dry weight was measured and the water loss calculated.

### 4.2. Free IAA Determination

The determination of free IAA was performed according to Ludwig-Müller et al. and Mittag et al. [15,16]. Briefly, the plant material was extracted with iso-propanol:acetic acid (95:5, *v/v*). To each sample 100 ng ^13^C_6_-IAA (Cambridge Isotope Laboratories, Andover, MS, USA) was added. For each line three independent extractions were performed. Methylation of all samples was carried out with trimethylsilyldiazomethane [85]. GC–MS analysis was carried out on a Varian Saturn 2100 ion-trap mass spectrometer using electron impact ionization at 70 eV, connected to a Varian CP-3900 gas chromatograph equipped with a CP-8400 autosampler (Varian, Walnut Creek, CA, USA). For the analysis 1 µL of the methylated sample was injected onto a 30 m ZB-5 column (Phenomenex, Darmstadt, Germany) using He carrier gas at 1 mL min^−1^. Injector temperature was 250 °C and the temperature program was 70 °C for 1 min, followed by an increase of 20 °C min^−1^ to 280 °C, then 5 min isothermically at 280 °C. For higher sensitivity, the µSIS mode was used. The settings of the MS were as previously described [86]. The endogenous concentrations of IAA were calculated according to the principles of isotope dilution [87] monitoring the quinolinium ions at m/z 130/136 (ions deriving from endogenous and ^13^C_6_-IAA, respectively).

### 4.3. Lipid Peroxidation Determination

The determination of the lipid peroxidation level was done by determination of a product of lipid peroxidation, malondialdehyde (MDA), content using the thiobarbituric acid (TBA) reaction according to Heath and Packer [88] with minor modifications. The MDA content was measured after 12, 24, and 36 days after transfer to salt-containing or control plates. From each sample 120 mg fresh weight was homogenized in 1 mL extraction solution, consisting of 0.1% trichloroacetic (TCA) and 1% sodium dodecyl sulfate (SDS) in distilled water. Homogenization was done in a cold mortar and with a pestle, using liquid N_2_. The homogenate was centrifuged at 4 °C at 15,000× *g* for 15 min. Of the supernatant 300 μL was added to 1 mL of the MDA reagent, including 20% TCA-SDS solution and 0.5% TBA. The mixture was incubated at 95 °C for 30 min and then cooled on ice. After centrifugation at 4 °C for 10 min, the absorbance of the supernatant was measured at 532 nm. The concentration of MDA was calculated using its extinction coefficient (εM) of 155 mM^−1^ cm^−1^. Each sample was repeated three times and finally the mean was expressed in nanomoles malondialdehyde per gram fresh weight (nmol MDA/g FW).

### 4.4. Total Phenol Determination

The total phenolic content of samples was determined 12, 24, and 36 days after transfer to NaCl or control plans of the same age using the Folin–Ciocalteu (FC) method [89]. From each sample 100 mg fresh weight was homogenized in 2mL 70% MeOH using mortar and pestle. The homogenates were kept at room temperature for 5–10 min under gentle shaking and were then centrifuged for 5 min at 1500× *g*. The supernatants were evaporated to the aqueous phase under a stream of nitrogen and the pH was adjusted to 3. Total phenols were extracted using two volumes of ethyl acetate and the samples were centrifuged at 1500× *g*. The procedure was repeated twice. The organic phases were carefully removed and collected after each centrifugation step and combined. The collected supernatants were evaporated to dryness under N_2_ and dissolved in 100 μL methanol. The total volume of the samples (plant and standards) was mixed with 500 μL FC reagent and 400 μL Na_2_CO_3_ and incubated for 2 h at room temperature in the darkness. The absorbance of triplicate samples was measured at 765 nm. To calculate the total phenolic content a standard curve of known concentrations of gallic acid (GA) was measured. For this, a 25 mg mL^−1^ stock solution of GA was made up by dissolving the respective concentration of GA (25, 50, 100, 150, and 200 mg/L) in methanol (95%). Each sample was repeated three times and the mean was expressed in mg GA g FW^−1^.

### 4.5. In Situ Staining of Flavonoids

The fluorescence of diphenylboric acid 2-amino-ethyl-ester (DPBA) conjugated to the flavonoid compounds, after excitation with blue light, was used to visualize the locations of the flavonoid compounds in situ according to Buer and Muday [50]. The staining was carried out using individual plants 12, 24, and 36 days after transfer to control and salt-containing plates. A 10% (*w/v*) stock solution of DPBA in 95% MeOH was prepared, which was diluted to 0.25% DPBA in distilled H_2_O containing 0.005% (*v/v*) Triton X-100. All samples were incubated for 15 min and then washed for 5 min in 100 mM sodium phosphate buffer, pH 7 (including 0.005% (*v/v*) Triton X-100, 0). Negative controls without DPBA reagent were prepared to confirm that the fluorescence colors came from the DPBA conjugates. After staining, the samples were placed on slides using 50% (*v/v*) glycerol. Fluorescence was visualized by excitation with blue radiation and the following filter (G365/FT395/LP420) on a Zeiss Axioskop 2 (Carl Zeiss, Jena, Germany) fluorescence microscope (at 366 nm the fluorescences of quercetin were yellow, of kaempferol green and of naringenin cyan).

### 4.6. Protein Extraction

The plant samples were prepared 12, 24, and 36 days after transfer to control and salt-containing plates according to a modified method of Qureshi et al. [90]. Ca 120 mg fresh weight of the respective plant material was homogenized in a cold mortar with a pestle and 350 μL cold extraction buffer of 100 mM potassium-phosphate buffer, pH 7.4, containing 1 mM ethylenediaminetetraacetic acid and 1 mM phenylmethylsulfonyl fluoride. After centrifugation at 15,000× *g* and 4 °C for 25 min, the total protein content of the supernatants was spectrophotometrically measured (see below) and the extracts were used for the enzyme assays described below.

### 4.7. Total Protein Determination

Total protein content was measured using the bicinchoninic acid (BCA) method according to the manufacturer’s instructions. The plant material was extracted with the respective buffers used for enzyme determination. When reducing agents were present, the proteins were precipitated with acetone (400 µL added to 200 µL protein solution, 30 min incubation at −20 °C, centrifugation for 5 min at 15,000× *g*) from the buffer solution. The preparation and assaying were according to the instructions for the BCA Protein Assay Reagent Kit (Thermo Fisher Scientific, Dreieich, Germany). The absorbance was measured at 562 nm. Different dilutions of Bovine Serum Albumin Standard (BSA) in the extraction buffer with the working range of 5–250 μg/mL were used to create a standard curve. All samples were measured in triplicates.

### 4.8. Peroxidase Enzyme Activity

In order to determine peroxidase activity, o-phenylenediamine (OPD) was used as substrate [54]. A protein extract (5 μg) was incubated for 15 min with 0.5 mL substrate buffer (one tablet of OPD in 5 mL 20 mM Tricine-KOH, pH 7.0, containing 3.6% H_2_O_2_). The substrate buffer should be freshly prepared and the tablets have to be dissolved fresh on the day of the experiment. After the incubation time, 0.5 mL 0.5 M H_2_SO_4_ was used to stop the reaction. The absorbance of the samples was determined at 490 nm. In order to calculate the enzyme activity, a defined amount of horseradish peroxidase was used for the preparation of the standard curve. For this aim, different dilutions of horseradish peroxidase in extraction buffer with the working range of 0.1–10 mU μL^−1^ were prepared. A standard curve was plotted based on their absorbance at 490 nm.

### 4.9. Native Polyacrylamide Gel Electrophoresis (PAGE)

The detection of the isoenzyme patterns for two different groups of enzymes was performed on a native poly-acrylamide gel electrophoresis in the presence of low amounts of SDS (0.1%) in the running buffer. No reducing agents were present [54]. For the detection of the enzyme activities, the electrophoresis was carried out at 4 °C. Total proteins were separated and the resulting patterns were stained for peroxidase (POX) and superoxide dismutase (SOD) activities and total protein (Coomassie). Equal amounts of protein (50 μg) were loaded in each lane of the gels. The resolving gel and stacking gel had acrylamide concentrations of 10% and 5%, respectively. A molecular mass marker (Thermo Fisher Scientific, Dreieich, Germany) was run on all gels along with the tested samples.

#### 4.9.1. Peroxidase Isoenzyme Detection

The detection of the peroxidase isoenzyme patterns was performed according to Ludwig-Müller et al. [54] using benzidine-guaiacol as substrates to visualize the bands. The staining solution comprised of solution A: 40 mL 0.2 M Na-acetate, 4 mL 5 mM MnSO_4_, 4 mL 0.35% H_2_O_2_ and solution B: 20 mg benzidine (4,4′-diaminobiphenyl), 10 mL 10% acetic acid, 54 μL guaiacol. When the run of the polyacrylamide gel was completed, the gel was quickly rinsed in H_2_O and then placed into solution A. Incubation was performed at room temperature. Under constant shaking, solution B was added to the gel. Staining was continued until the POX bands were visible on the gel. The reaction was stopped by replacing the staining solution several times with water. The density of the bands was detected and displayed using the GelEval software free trial version 1.35 (FrogDanceSoftware, Cambridge, UK).

#### 4.9.2. Superoxide Dismutase Isoenzyme Detection

SOD isoenzymes were detected in the gels by their ability to inhibit the photochemical reduction of nitroblue tetrazolium (NBT). The staining of the gels was performed based on the method described by Pitzschke et al. [55] with some modifications. The gels were rinsed in cold distilled water, then incubated for 20–25 min in 2 mM NBT (made in 100 mM potassium phosphate buffer, pH 7.8) under constant agitation in light at 4 °C. The NBT solution was then replaced with riboflavin solution [45] (0.030 mM riboflavin, 1% N, N, N′, N′-tetramethylethylenediamine (TEMED) in the same buffer) and the gels were further incubated for 25–30 min at 4 °C in darkness. Finally, the gels were briefly rinsed in distilled water and SOD activity was shown by white bands against a violet background.

#### 4.9.3. Total Protein Staining with Colloidal Commassie

The total protein pattern was determined using the colloidal Coomassie staining method based on the protocol of Neuhoff et al. [91] with some modifications. The gel was incubated for 20 min in 50 mL solution A (10% (*w/v*) ammonium sulfate, 2% phosphoric acid in distilled H_2_O) containing 1.25 mL solution B (5% (*w/v*) Coomassie Brilliant Blue G250 in distilled H_2_O) under continuous shaking. The gel was then destained in 25% methanol in distilled H_2_O followed by destaining in methanol until the background was clear.

### 4.10. Statistical Analysis

The results of the experiments were analyzed using the “IBM SPSS Statistics 20” program. Data are the mean values of at least three replicates. The error bars shown in all bar graphs represent standard deviation calculated from all repetitions of each experiment. Analysis of independent data was done by the two-way ANOVA (univariate) method and the significance of differences was determined using the Tukey test. The test of a Repeated Measurement ANOVA was carried out in order to analyze the dependent data [92,93]. Differences at the level of *p* ≤ 0.05 were considered significant.

### 4.11. Digital Expression and Promoter Element Analyses

Digital transcription analysis with the annotated gene numbers for *PpGH3.1* (Pp3c24_16260V3.1/Pp1s323_82V6.1) and *PpGH3-2* (Pp3c10_20960V3.1/Pp1s67_243V6.1), the numbers depend on the annotation version, as a query was used to analyze the transcription levels under various stress conditions using available web resources (Phytozome, GeneAtlas) [41,42,43,44]. The promoter sequences of both *GH3* genes (circa 3 kb upstream of the transcription start in the genome sequence) was analyzed for the occurrence of possible regulatory elements taken from Phytozome [41]).

## 5. Conclusions

In conclusion, our results show that selected parameters connected to oxidative stress are differentially regulated in *P. patens* after exposing gametophore tissue to high salt concentrations (250 mM), but that these was not dependent upon the auxin conjugating GH3 proteins. However, the auxin conjugate formation catalyzed by the GH3 proteins seems to be involved in stress response regulation since two of the double KO mutants were more resistant to high salt concentrations. The results indicate the delicate balance between growth and stress adaptation through the regulation of free auxin levels since the double KO mutant lines showed a growth inhibition under normal conditions and at high auxin levels but grew better in high salt conditions. However, the high auxin levels in the mutant lines presumably did not confer the induction of other stress responses.

## Data Availability

All data supporting this study are available in this paper and in its supplementary data published online.

## Supplementary Materials

The following are available online at https://www.mdpi.com/article/10.3390/plants10071398/s1, Figure S1: Wild type and 4 independent GH3 double-KO mutants of *P. patens* under control and at different salt concentrations over time. Figure S2: Fresh weight and growth rate for wild type and 4 independent GH3 double-KO mutants of *P. patens*. Figure S3: Fresh weight and dry weight to calculate the water loss in the tissue for wild type and 2 independent GH3 double-KO mutants of *P. patens*. Figure S4: Total protein content, lipid peroxidation, total phenol content, and peroxidase activity in wild type and 4 independent GH3 double-KO mutants of *P. patens*. Figure S5: KEGG pathway for flavonoids in *P. patens*. Figure S6: In situ flavonoid staining in wild type and 4 independent GH3 double-KO mutants of *P. patens*. Figure S7: The original PAGE gels for total protein stained with colloidal Coomassie blue (**A**), peroxidase isoenzyme separation (**B**) and superoxide dismutate (SOD) isoenzyme separation (**C**) for wild type and four independent GH3 double-KO mutants of *P. patens*. Table S1: Analysis of stress and stress hormone related elements in the promoter of the two *P. patens GH3* genes. Table S2: The most strongly co-expressed genes for *PpGH3-1* and *PpGH3-2*.
