# Supplementary material for: Physcomitrium patens Mutants in Auxin Conjugating GH3 Proteins Show Salt Stress Tolerance but Auxin Homeostasis Is Not Involved in Regulation of Oxidative Stress Factors
Source: Plants (Basel). 2021 Jul 8;10(7):1398. doi: 10.3390/plants10071398 (PMC8309278; doi:10.3390/plants10071398)
Supplement: Supplementary file 1 [file plants-10-01398-s001.zip › plants-1253879-supplementary.pdf]

Supplement to

Physcomitrium patens Mutants in Auxin Conjugating GH3 Proteins Show Salt Stress Tolerance but Auxin Homeostasis is not Involved in Regulation of Oxidative Stress Factors

Haniyeh Koochak and Jutta Ludwig-Müller

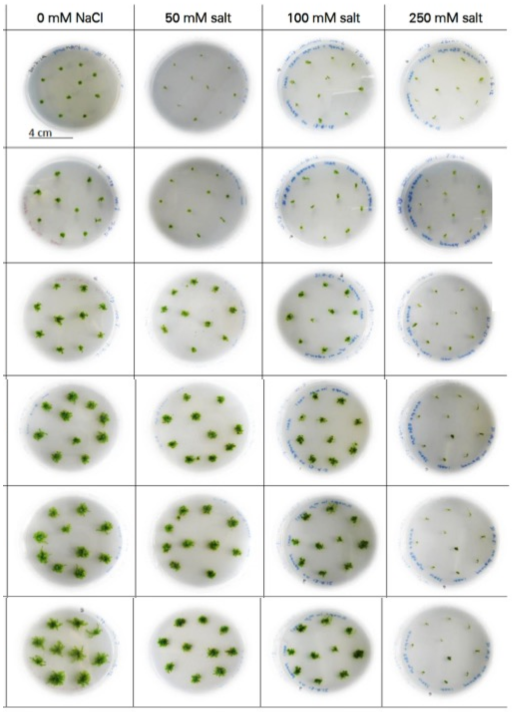

WT

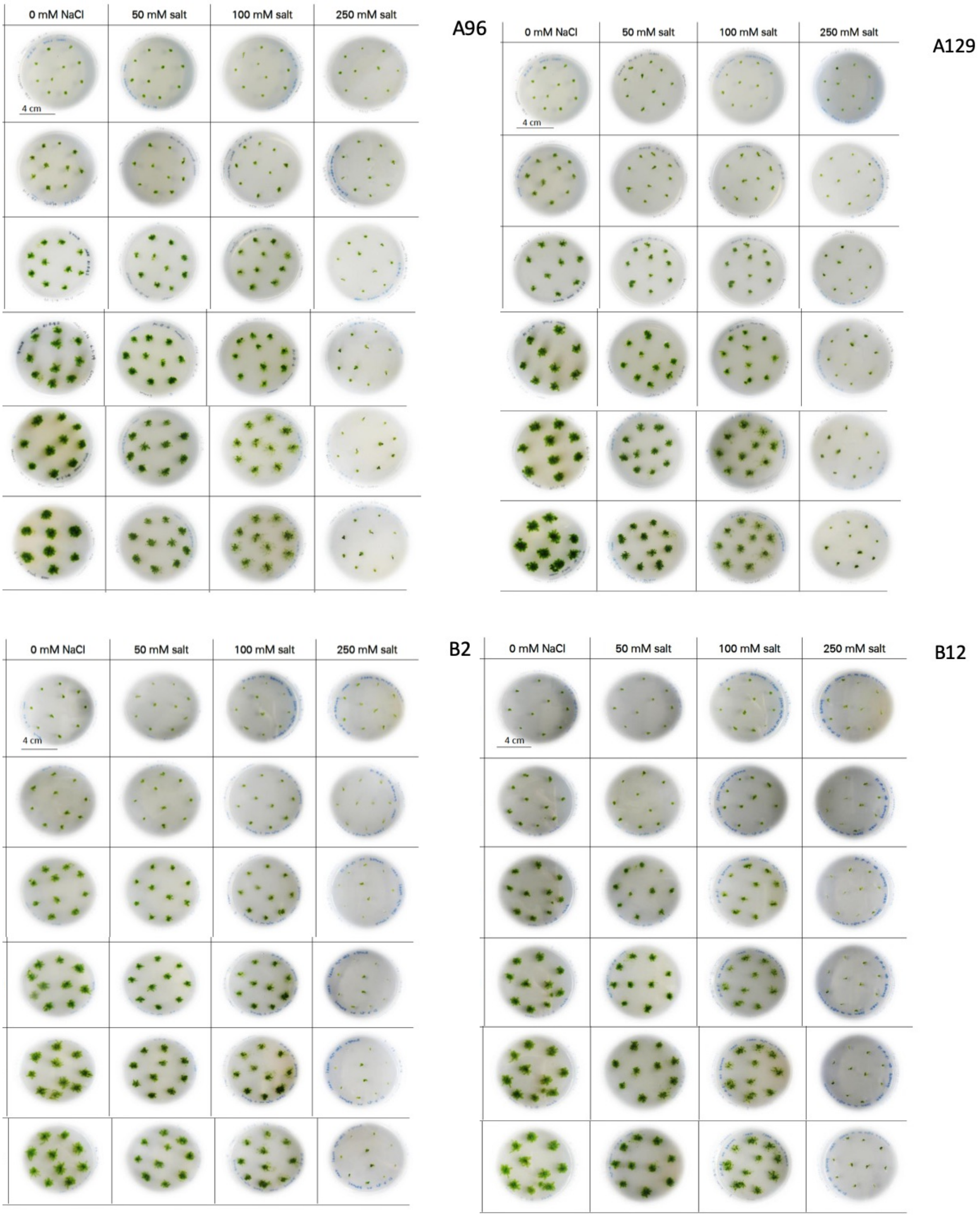

Figure S1. Wild type and 4 independent GH3 double-KO mutants of *P. patens* on control, 50, 100 and 250 mM salt (NaCl) at 4, 14, 24, 33, 42, and 50 (from top to bottom) days after transfer from control KNOP medium to experimental medium.

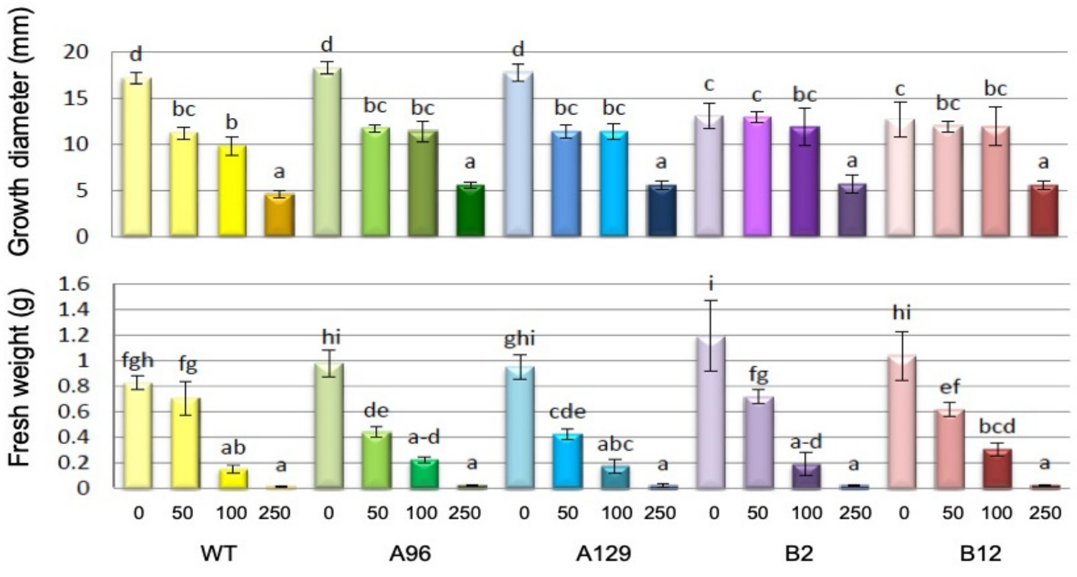

Figure S2. Fresh weight and growth rate for wild type and 4 independent GH3 double-KO mutants of *P. patens* on control, 50, 100 and 250 mM salt (NaCl) 50 days after transfer from control KNOP medium to experimental medium. Bars with different letters are significantly different at $p \leq 0.05$.

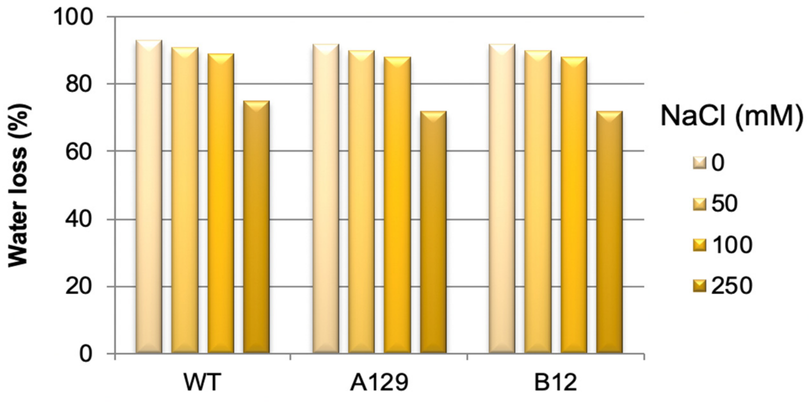

Figure S3. Fresh weight and dry weight were used to calculate the water loss in the tissue for wild type and 2 independent GH3 double-KO mutants of *P. patens* on control (0), 50, 100 and 250 mM salt (NaCl) 36 days after transfer from control KNOP medium to experimental medium. No significant differences between wild type and mutant lines were found.

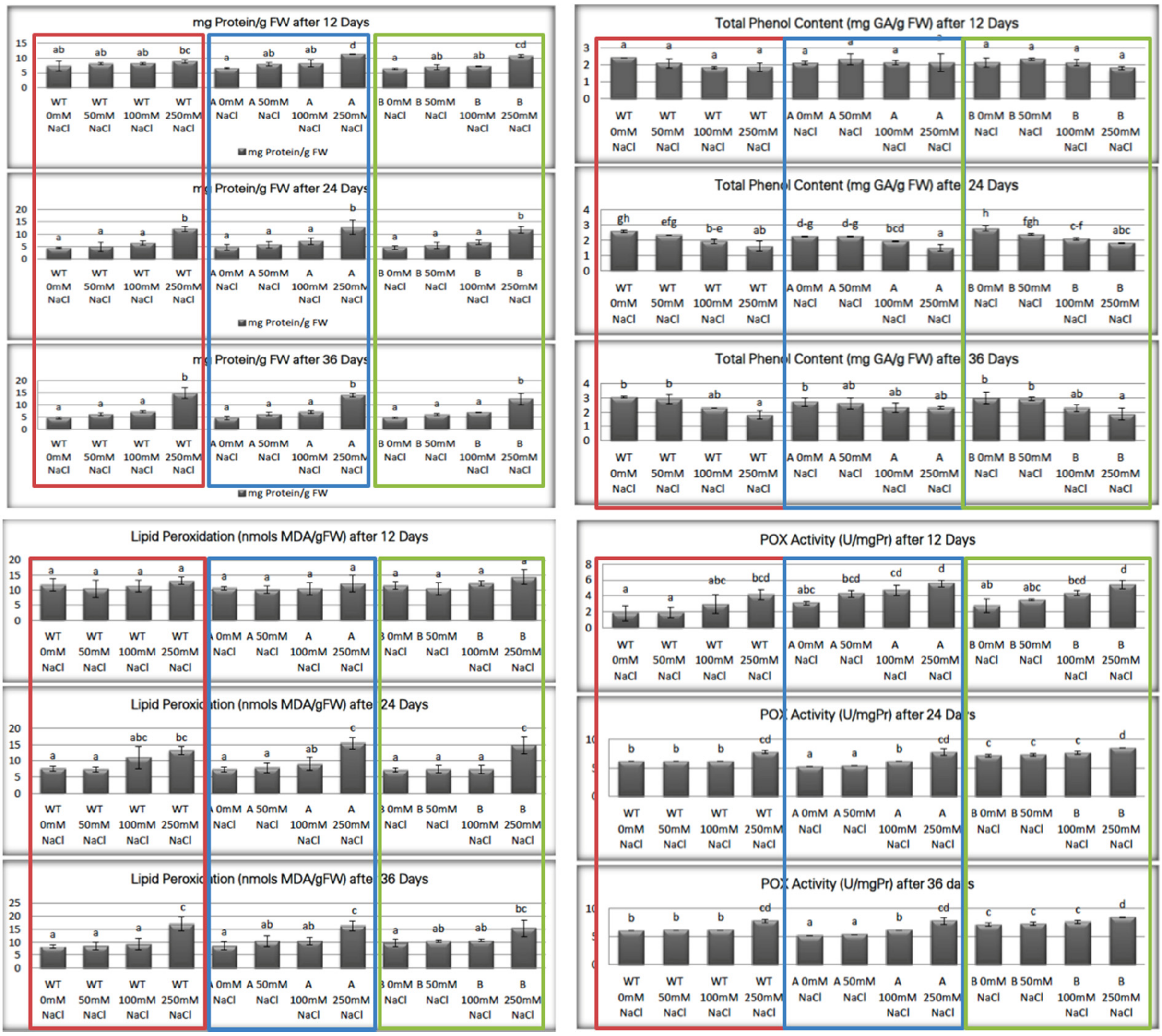

Figure S4. Total protein content, lipid peroxidation, total phenol content, and peroxidase activity in wild type (red frame) and 4 independent GH3 double-KO mutants (A mutant lines blue frame; B mutant lines green frame) of *P. patens* on control, 50, 100 and 250 mM salt (NaCl) at 12, 24, and 36 days after transfer from control KNOP medium to experimental medium. Bars with different letters are significantly different at $p \leq 0.05$. Selected values where differences can be found are shown in the main manuscript.

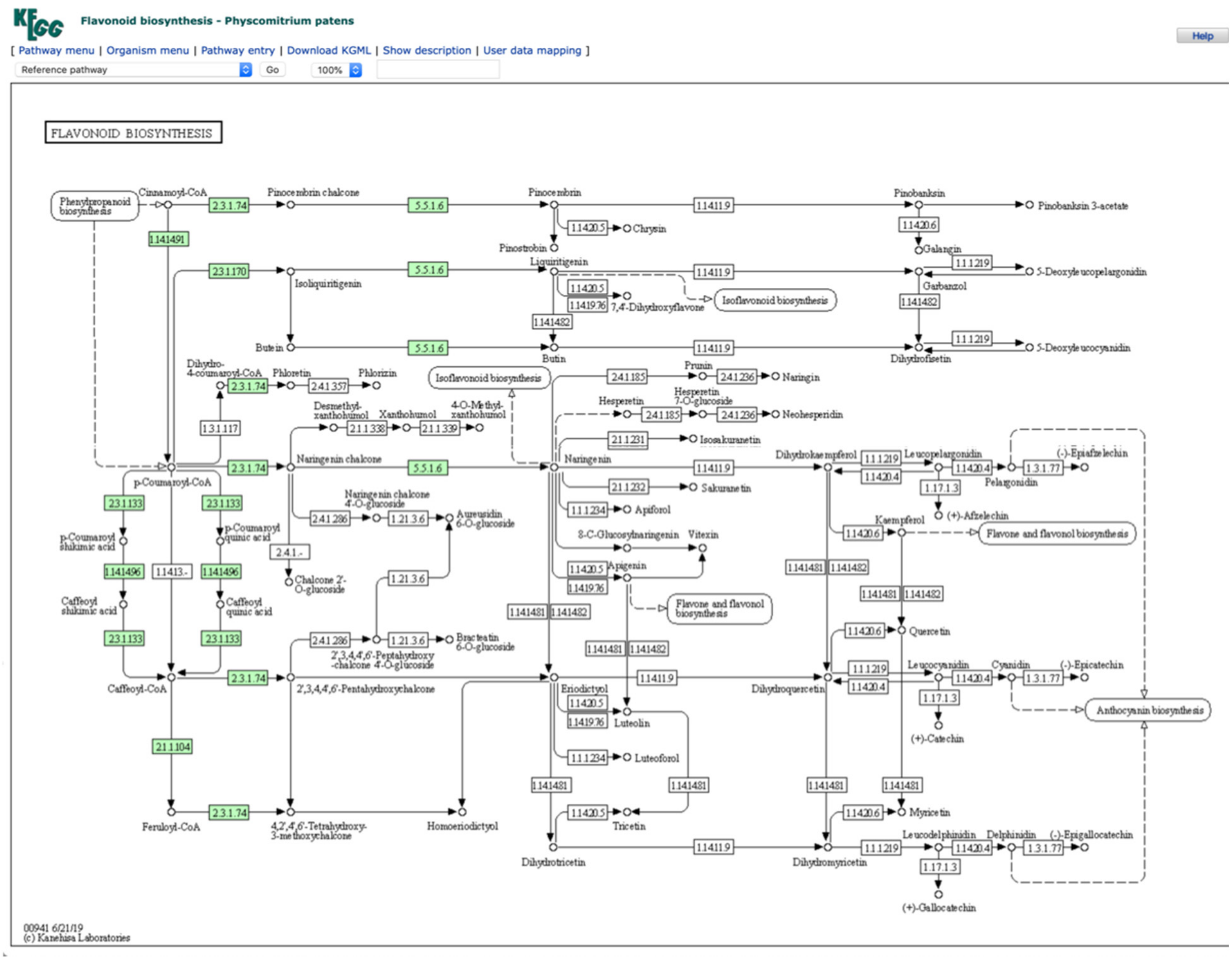

Figure S5. KEGG (Kyoto Encyclopedia of Genes and Genomes) pathway for flavonoid biosynthesis of *Physcomitrium patens*.

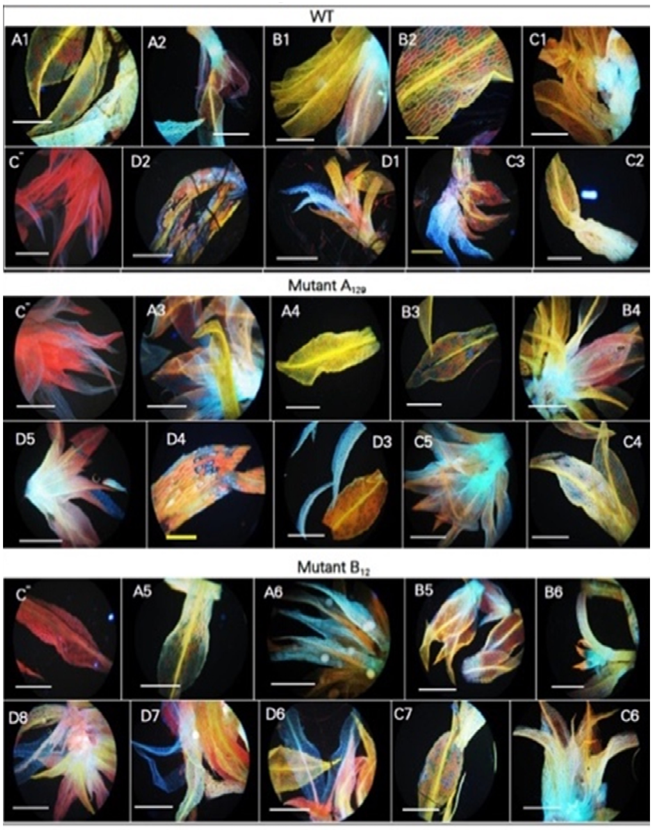
WT
A1
A2
B1
B2
C1
C⁻
D2
D1
C3
C2
Mutant A_{129}
C⁻
A3
A4
B3
B4
D5
D4
D3
C5
C4
Mutant B_{12}
C⁻
A5
A6
B5
B6
D8
D7
D6
C7
C6

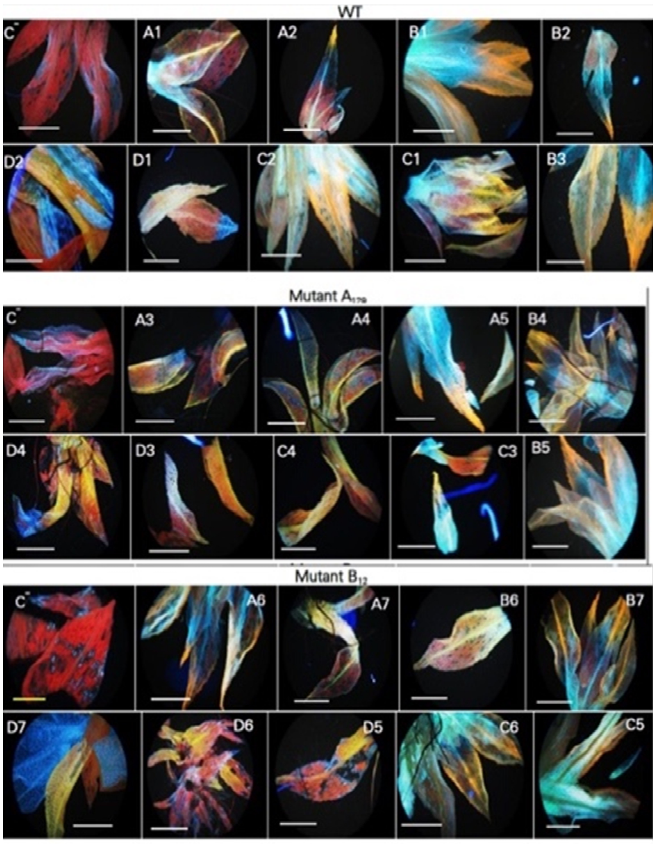
WT
C⁻
A1
A2
B1
B2
D2
D1
C2
C1
B3
Mutant A_{129}
C⁻
A3
A4
A5
B4
D4
D3
C4
C3
B5
Mutant B_{12}
C⁻
A6
A7
B6
B7
D7
D6
D5
C6
C5

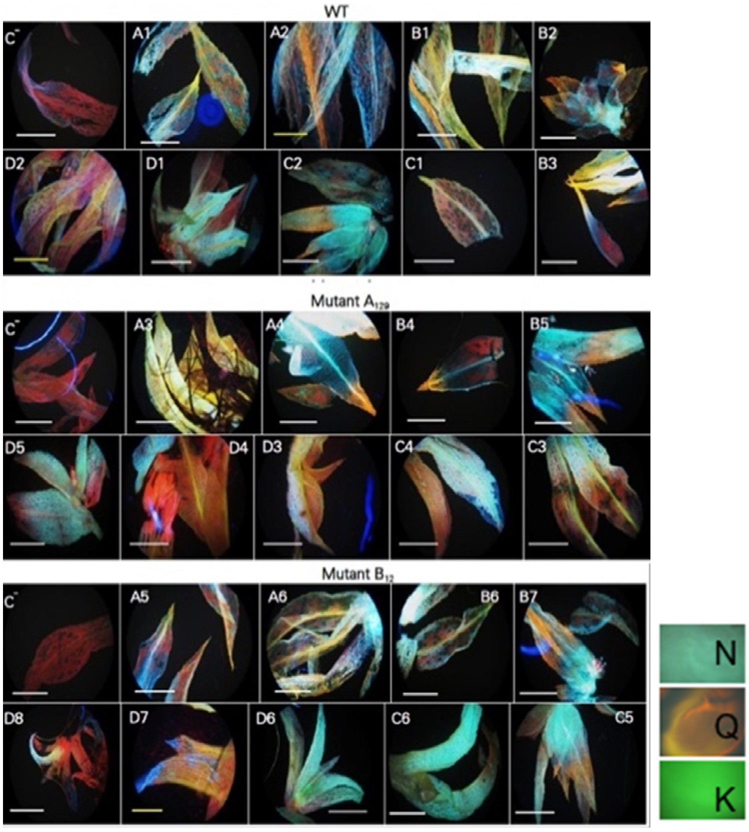

Figure S6. In situ flavonoid staining in wild type and 4 independent GH3 double-KO mutants of *P. patens* on control (A), 50 (B), 100 (C) and 250 mM (D) salt (NaCl) at 12 (top panel combination), 24 (Middle panel combination, and 36 (bottom panel combination) days after transfer from control KNOP medium to experimental medium. For all conditions two photos are shown. The first panel of each picture group (C^-) denotes the control stain without DPBA showing the red autofluorescence of chlorophylls. White and yellow bars represent 1 and 100 µM, respectively. The colors indicating the different flavonoids (taken from Päsold, S.; Siegel, I.; Seidel, C.; Ludwig-Müller, J. Flavonoid accumulation in *Arabidopsis thaliana* root galls caused by the obligate biotrophic pathogen *Plasmodiophora brassicae*. *Mol. Plant Pathol.*, **2010**, *11*, 545-562.) are shown in the bottom panel.

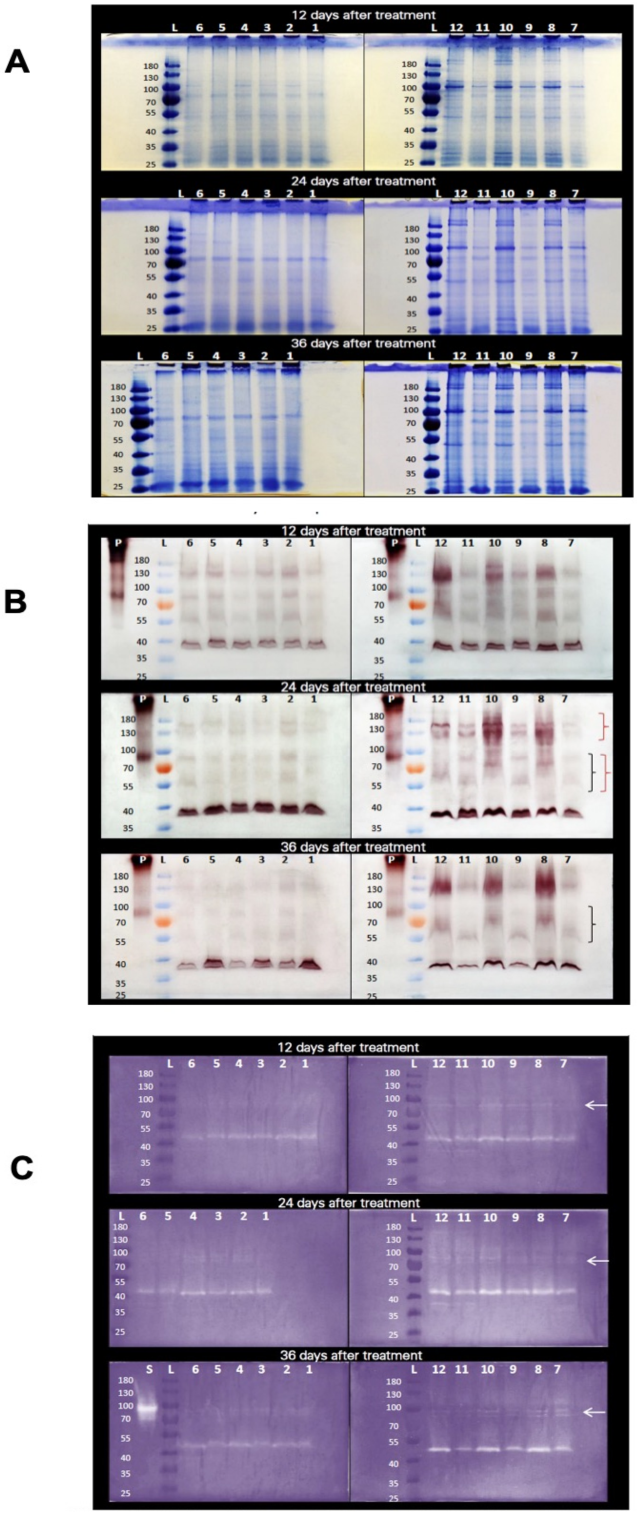

Figure S7. The original PAGE gels for total protein stained with Colloidal Coomassie Blue (A), peroxidase isoenzyme separation (B) and superoxide dismutate (SOD) isoenzyme separation (C) for wild type and 4 independent GH3 double-KO mutants of *P. patens* on control, and different salt (NaCl) concentrations at 12, 24, and 36 days after transfer from control KNOP medium to experimental medium. Bars with different letters are significantly different at $p \leq 0.05$. Selected values where differences can be found are shown in the main manuscript. L = marker; P = horseradish peroxidase standard; S = SOD standard. Lanes 1,2,7,8: wild type at 0, 50, 100, 250 mM NaCl; lanes 3,4,9,10: mutant line A129 at 0, 50, 100, 250 mM NaCl; lanes 5,6,11,12: mutant line B12 at 0, 50, 100, 250 mM NaCl.

Table S1. Analysis of stress and stress hormone related elements in the promoter of the two *P. patens GH3* genes. Elements in a region of 3 kb upstream of the transcription start are given.

**Function of element**	**pGH3-1**	**pGH3-2**
Abscisic acid responsive element	ABRE (3) [1]	--
Abscisic acid responsive element	CE3 (1)	--
Ethylene responsiveness	--	ERE (1)
Gibberellin responsiveness	GARE (1)	GARE (2)
Methyl-Jasmonate responsiveness	CGTA motif (4)	--
Methyl-Jasmonate responsiveness	TGACG motif (4)	--
Salicylic acid responsiveness	TCA element (2)	--
Drought inducibility (Myb binding site)	MBS (2)	MBS (1)
Involvement in defense and stress responsiveness	TC-rich element (1)	TC-rich repeat (1)
Heat shock element	--	HSE (2)
Low temperature responsiveness	--	LTR (1)

[1] The numbers in brackets denote the total number of this element within the promoter sequence.

Table S2. The most strongly co-expressed genes for *PpGH3-1* and *PpGH3-2* found in databases. In red are annotations for stress- and hormone-related genes shown. Data were obtained from the Gene Atlas (Phytozome) [27, 28] with the two *PpGH3* genes as query.

**gene number**	**coefficient**	**description**
PpGH3-1		
Pp3c4_18780	0.916	(M=31) 2.7.10.1//2.7.11.1 - Receptor protein tyrosine kinase / Receptor protein tyrosine kinase // Non-specific serine/threonine protein kinase / Threonine-specific protein kinase
Pp3c23_17090	0.91	(M=736) 2.7.11.1 - Non-specific serine/threonine protein kinase / Threonine-specific protein kinase
Pp3c2_33610	0.91	(M=13) PTHR31439:SF2 - GB
Pp3c14_17980	0.908	(M=1) PTHR10314:SF21 - TRYPTOPHAN SYNTHASE BETA CHAIN
Pp3c18_14230	0.906	(M=3) PTHR11062:SF64 - EXOSTOSIN FAMILY PROTEIN-RELATED
Pp3c1_16500	0.906	(M=2) PTHR30249 - PUTATIVE SEROTONIN TRANSPORTER
Pp3c14_10390	0.901	(M=6) PTHR24351:SF81 - PHOTOTROPIN-2
Pp3c14_12030	0.9	(M=10) PTHR31052:SF3 - COBRA-LIKE PROTEIN 7-RELATED
Pp3c11_1840	0.897	(M=38) PF03106 - WRKY DNA -binding domain (WRKY)
Pp3c11_14370	0.896	(M=9) 5.1.3.6 - UDP-glucuronate 4-epimerase / Uridine diphosphoglucuronate epimerase
Pp3c1_11030	0.895	(M=3) 2.7.11.18 - [Myosin light-chain] kinase / Smooth-muscle-myosin-light-chain kinase
Pp3c24_17380	0.895	(M=4) PTHR10314:SF125 - THREONINE SYNTHASE 2, CHLOROPLASTIC
PpGH3-2		
Pp3c25_700	0.958	(M=3) 2.3.2.2//2.6.1.2//2.6.1.4//2.6.1.44 - Gamma-glutamyltransferase / Glutamyl transpeptidase // Alanine transaminase / Glutamic--pyruvic transaminase // Glycine transaminase / Glycine aminotransferase // Alanine--glyoxylate transaminase / Alanine--glyoxylate aminotransferase
Pp3c16_15270	0.953	(M=3) PF00430 - ATP synthase B/B' CF(0) (ATP-synt_B)
Pp3c4_10300	0.951	(M=1) PTHR34051:SF2 - PROTEIN LOW PSII ACCUMULATION 3, CHLOROPLASTIC
Pp3c10_380	0.949	(M=3) KOG0855 - Alkyl hydroperoxide reductase, thiol specific antioxidant and related enzymes
Pp3c24_12360	0.948	(M=3) PTHR12652:SF17 - PEROXISOMAL MEMBRANE PROTEIN 11B
Pp3c3_32790	0.945	(M=2) PTHR35753:SF1 - PROLINE-RICH FAMILY PROTEIN
Pp3c5_10250	0.945	(M=3) 2.3.2.2//2.6.1.2//2.6.1.4//2.6.1.44 - Gamma-glutamyltransferase / Glutamyl transpeptidase // Alanine transaminase / Glutamic--pyruvic transaminase // Glycine transaminase / Glycine aminotransferase // Alanine--glyoxylate transaminase / Alanine--glyoxylate aminotransferase
Pp3c4_25980	0.942	(M=3) PTHR11584:SF316 - SERINE/THREONINE-PROTEIN KINASE STN7, CHLOROPLASTIC
Pp3c14_19950	0.938	(M=8) PF09353 - Domain of unknown function (DUF1995) (DUF1995)
Pp3c10_10490	0.937	(M=3) PTHR11177:SF167 - RHODANESE-LIKE DOMAIN-CONTAINING PROTEIN 4, CHLOROPLASTIC
Pp3c4_8159	0.935	(M=4) KOG3309 - Ferredoxin
Pp3c11_8540	0.933	(M=1) PTHR13789//PTHR13789:SF216 - MONOOXYGENASE
